# Severe level of photochemical oxidants (O_x_) over the western coast of Japan during autumn after typhoon passing

**DOI:** 10.1038/s41598-023-43485-0

**Published:** 2023-09-29

**Authors:** Syuichi Itahashi

**Affiliations:** Sustainable System Research Laboratory (SSRL), Central Research Institute of Electric Power Industry (CRIEPI), Abiko, Chiba 270-1194 Japan

**Keywords:** Environmental chemistry, Atmospheric chemistry, Atmospheric dynamics

## Abstract

Photochemical oxidants (O_x_; mainly O_3_) are a concern in East Asia. Because of the prevailing westerly wind in the midlatitudes, O_3_ concentration generally shows a high in spring over Kyushu Island, western Japan, and O_x_ warnings have been issued in spring. However, the record from 2000 to 2021 of O_x_ warning days in Kyushu Island contains one warning case in autumn 2020. Interestingly, a typhoon had passed the day before this O_x_ warning. To relate these events, a modelling simulation was conducted and it showed the transboundary O_3_ transport from the Asian continent to the western coast of Japan due to the strong wind field determined by the location of Typhoon Haishen (2020). The sensitivity simulations for changing Chinese anthropogenic sources suggested that both nitrogen oxides (NO_x_) and volatile organic compound (VOC) emission regulations in China could decrease high O_3_ over the downwind region of Japan. Furthermore, VOC emission regulation in China led to an overall O_3_ decrease in East Asia, whereas NO_x_ emission regulation in China had complex effects of decreasing (increasing) O_3_ during the daytime (nighttime) over China. The association between air quality and meteorology related to typhoons should be considered along with global warming in the future.

## Introduction

Photochemical oxidants (O_x_), which mainly consist of tropospheric ozone (O_3_), are related to chemical reactions involving nitrogen oxides (NO_x_) and volatile organic compounds (VOCs)^1^. O_x_ causes urban smog, poses major risks to human health and the natural environment, and is an important greenhouse gas^2^. The Japanese environmental quality standard (EQS) for O_x_ was established in 1973, and hourly O_x_ values should not exceed 0.06 ppm (118 μg/m^3^)^3^. In addition, the Air Pollution Control Law (Paragraph 1, Article 23) stipulates that when hourly O_x_ concentration exceeds 0.12 ppm and the status is expected to continue due to weather conditions, a warning should be issued to prevent damage to human health or the living environment. The EQS has not been satisfied at most monitoring stations in Japan, and this is a continuing issue that should be solved^4,5^. In general, high O_x_ concentrations and subsequent O_x_ warnings have generally occurred in spring over western Japan and in summer toward eastern Japan (e.g., Osaka, Nagoya, and Tokyo). This seasonal variation of O_x_ in Japan is related to the transboundary O_x_ transport from the Asian continent during spring and local photochemical production in Japan during summer^6–11^. A recent report on Japanese local photochemical production found a decreasing trend in the 3-year average of the annual 99^th^ percentile of the daily maximum 8-h concentration (a new index for evaluating O_x_), especially over the Kanto region (e.g., Tokyo)^12^, and this result suggested that transboundary O_x_ pollution has an important effect over Japan. The worsening of O_x_ pollution in East Asia has been a concern recently^13–15^; thus, the effect of transboundary transport on O_x_ behaviour is the focus of the present work.

**Figure 1:**
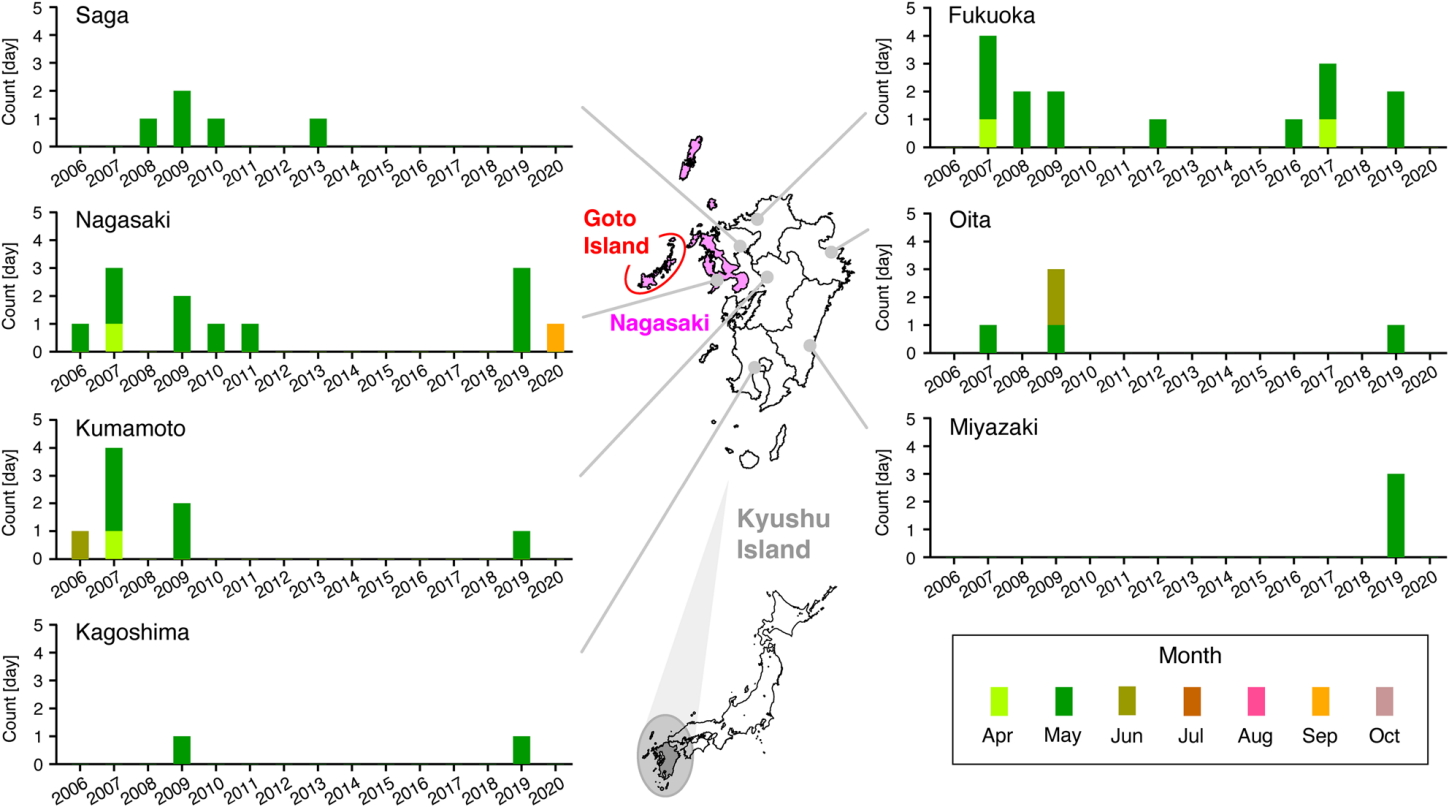
Record of the O_x_ warning days for severe concentrations (see, text for this definition) from 2000 to 2021 over seven prefectures on Kyushu Island, Japan. The colours indicate the month. At the start of this study, the confirmed values of AEROS were reported up to 2021^12^, and no days were reported in 2000–2005 and 2021 over all prefectures in Kyushu Island. The maps were generated with GMT (https://docs.generic-mapping-tools.org/dev/index.html).

Over Kyushu Island, western Japan, high O_x_ concentrations and related warnings are generally issued during spring. The summary of the number of O_x_ warning days from 2000 to 2021 over Kyushu Island is presented in Fig. 1. The O_x_ warning days have mostly been observed in May, and sometimes in April and June. In Nagasaki Prefecture, located on western Kyushu Island, an O_x_ warning was issued for the first time in May 2006. However, there was one warning day in September 2020 in Nagasaki Prefecture (Fig. 1). This warning was issued on Goto Island (westernmost Nagasaki Prefecture, Fig. 1) on 8 September, 2020 at 15:00 local time when an O_x_ concentration of 123 ppb was observed, the maximum concentration was recorded as 126 ppb at 16:00 local time, and the warning was canceled at 19:00 local time. In the present study, this severe O_x_ concentration event was analysed because this is the first case of a warning issued in autumn in Nagasaki Prefecture.

## Results

First, the meteorological conditions in September 2020 were analysed. Typhoons Maysak (2020) and Haishen (2020) occurred in this period^16^. Typhoon Maysak (2020) formed on 27 August, 2020 over the eastern Philippines and passed over western Kyushu Island on 2–3 September, and Typhoon Haishen (2020) formed on 31 August, 2020 over the Northwest Pacific and passed over western Kyushu Island on 6–7 September (Supplementary Fig. S1). The O_x_ warning on Goto Island in September 2020 was issued the day after Typhoon Haishen (2020) passed. Typhoons are usually associated with hazards, such as strong winds, storm surges, and rainfall, that can cause considerable damage and disruption. Although such a strong wind field could simply result in good ventilation and reduce air pollutant concentrations, several studies have reported that typhoons degraded air quality in terms of PM_2.5_^17,18^, O_x_^19,20^, and deposition^21,22^. Because the O_x_ warning issued in September 2020 occurred in a remote area of Goto Island, westernmost Nagasaki Prefecture, the horizontal distribution over East Asia should also be examined. Thus, a numerical modelling simulation covering the whole of East Asia (Supplementary Fig. S2) was applied to analyse this severe O_x_ event further (see “Methods” section for details of the modelling).

**Figure 2:**
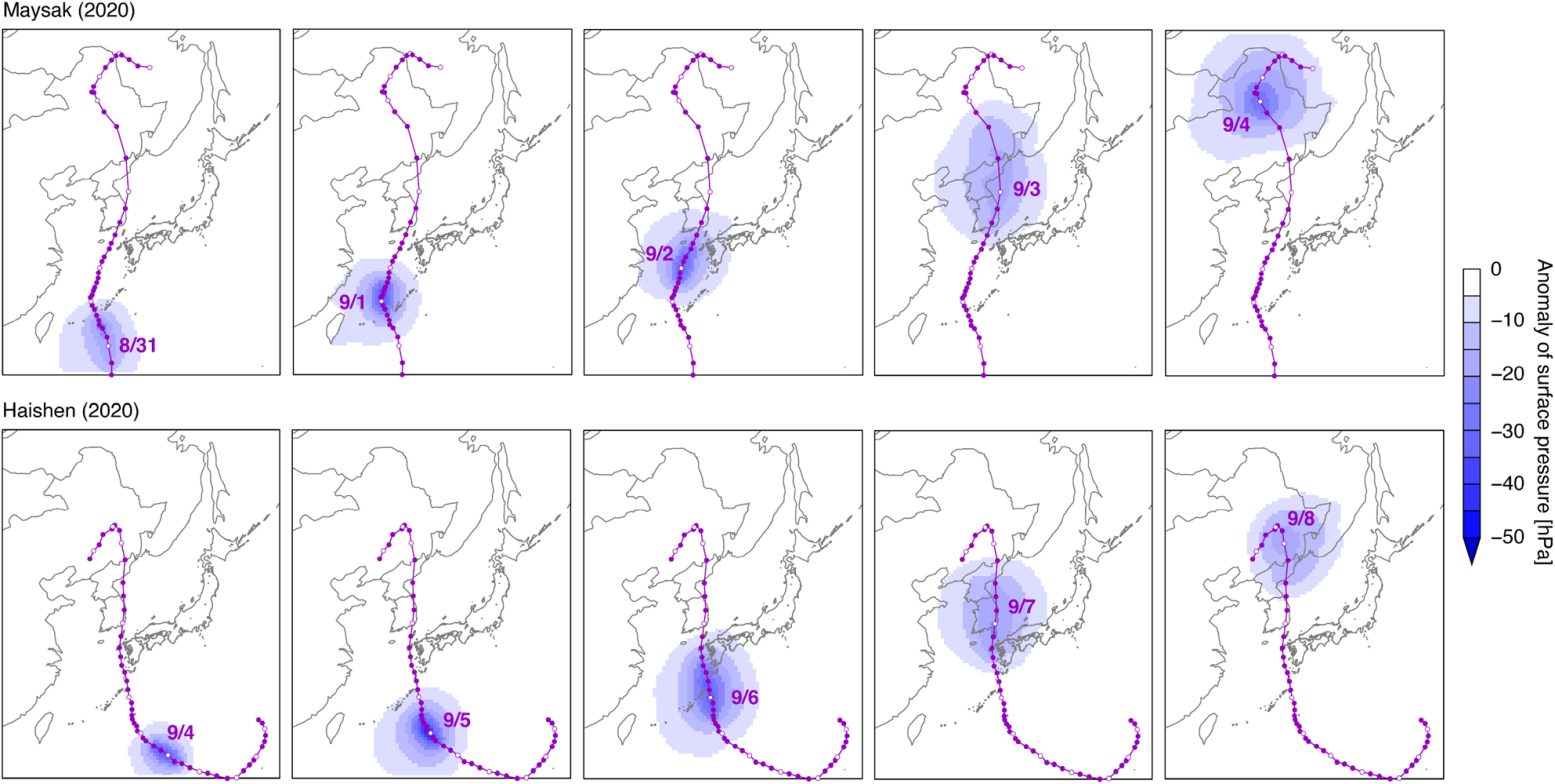
Best-track data of (top) Typhoon Maysak (2020) and (bottom) Typhoon Haishen (2020) with surface pressure anomalies simulated by the WRF meteorological model. The maps were generated with GMT (https://docs.generic-mapping-tools.org/dev/index.html).

The surface pressure anomaly, which is a unique characteristic of typhoons, was investigated and evaluated with the typhoon best-track data^16^. The typhoon tracks (Supplementary Fig. S1) and the simulated surface pressure anomaly are shown in Fig. 2. In this comparison, the anomaly was calculated by the difference between the surface pressure during each typhoon and the 1-month average (from 15 August, 2020 to 14 September, 2020; see “Methods” section). The typhoon best-track data and the simulated lower anomaly associated with Typhoons Maysak (2020) and Haishen (2020) agreed well. The comparison shown in Fig. 2 indicated that the general features of typhoon movement were captured well in the present modelling simulations.

**Figure 3:**
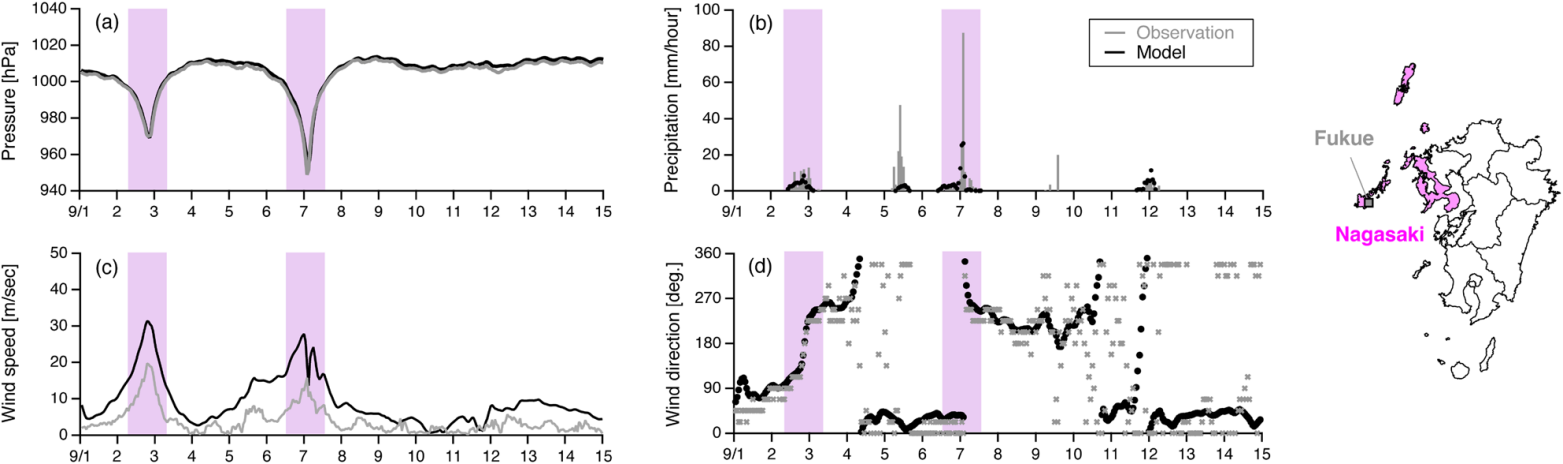
Comparison of meteorological fields of (**a**) pressure, (**b**) precipitation, (**c**) wind speed, and (**d**) wind direction from observations (grey) and the model (black) at Fukue, AMeDAS station in Nagasaki Prefecture. The purple shading indicates the days affected by Typhoon Maysak (2020) and Typhoon Haishen (2020). The maps were generated with GMT (https://docs.generic-mapping-tools.org/dev/index.html).

The meteorological parameters were measured by the Automated Meteorological Data Acquisition System (AMeDAS) at the Fukue observatory, which is on Goto Island (Fig. 1), and the corresponding simulated results are shown in Fig. 3. The pressure (Fig. 3a) was simulated well, and the decrease in pressure was larger in the period when Typhoon Haishen (2020) passed compared with that when Typhoon Maysak (2020) passed. The precipitation (Fig. 3b) was generally underestimated but the timing was well captured. The wind speed (Fig. 3c) and direction (Fig. 3d) were also well captured, although the wind speed tended to be overestimated. This overestimation was mainly due to the insufficient wind reduction over the land. In addition to the case at Fukue in Fig. 3, another validation at Nomozaki, southern Nagasaki Prefecture is presented in Supplementary Fig. S3. The Nomozaki site observed a record-breaking maximum instantaneous wind velocity (59.4 m/s, the corresponding wind velocity in 1 h was 43.7 m/s) during Typhoon Haishen (2020), and such wind speeds were generally captured by the modelling system. These evaluations of the meteorological parameters showed that the present modelling system reproduced the meteorological field well, especially the features when the typhoons passed.

**Figure 4:**
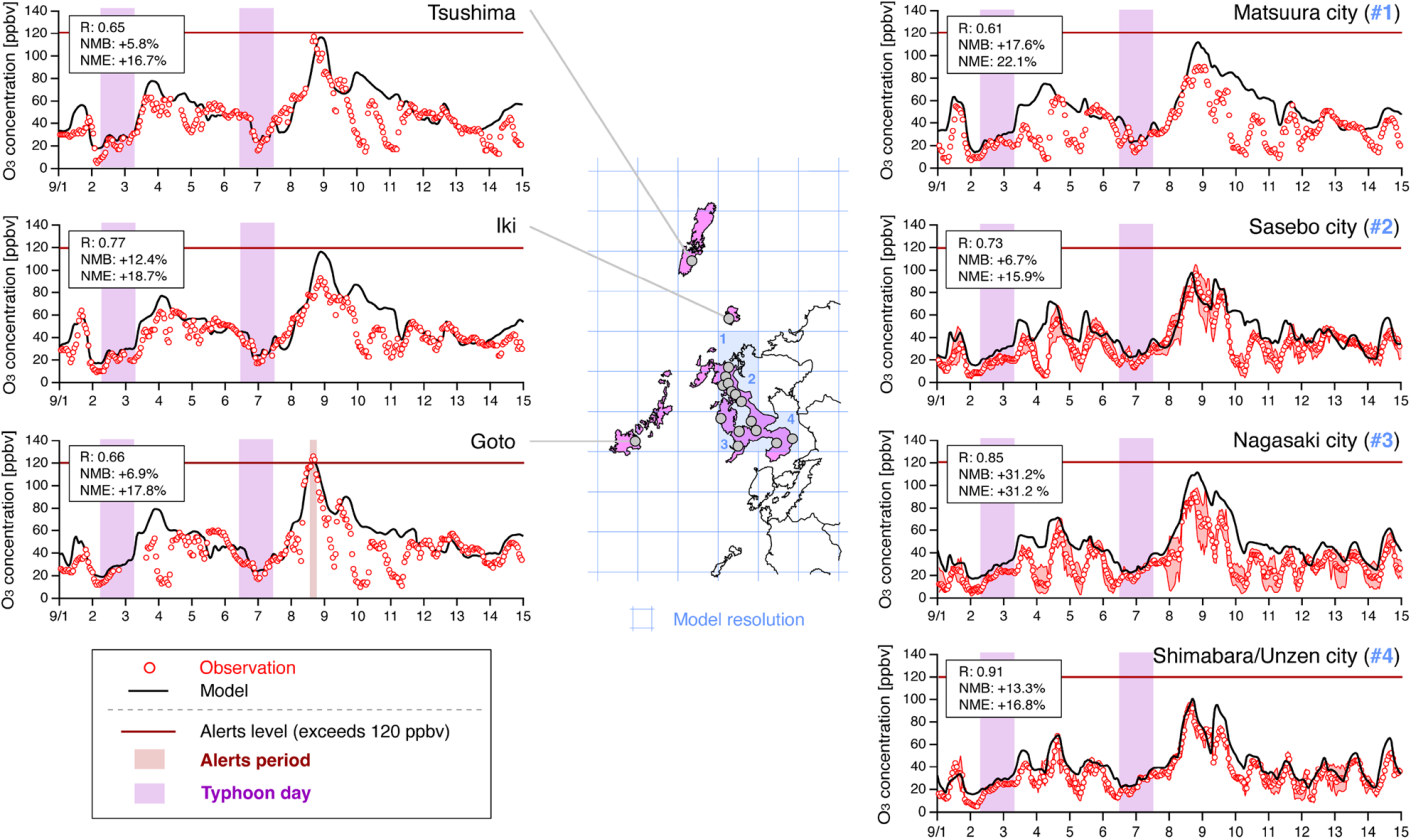
Comparison of O_3_ concentrations from observations (red) and the model (black) at all 17 sites in Nagasaki Prefecture. The purple shading indicates the days affected by typhoons (Fig. 3). The dark-red shading indicates the period of O_x_ alerts. When the measurement sites were allocated in the same model grid, the averaged values were used to evaluate the model, and the minimum and maximum concentrations are indicated by red shading. The statistical scores are shown in the box. The maps were generated with GMT (https://docs.generic-mapping-tools.org/dev/index.html).

The modelling performance for O_3_ concentration in Nagasaki Prefecture is shown in Fig. 4. The O_3_ concentration was lower during two typhoon periods (purple shading, see also Fig. 3). After Typhoon Maysak (2020) passed on 3 September, 2020, the O_3_ concentration was slightly increased to 60–80 ppbv at all sites in Nagasaki Prefecture. Subsequently, due to Typhoon Haishen (2020) passing, O_3_ concentration was decreased to 20 ppbv on 7 September, 2020. There was a sharp increase in O_3_ concentration after Typhoon Haishen (2020) passed, and the O_3_ concentration reached its peak from 8 to 9 September, 2020. This maximum concentration was close to 100 ppbv in cities in the main parts of Nagasaki Prefecture, whereas on the remote islands of Nagasaki Prefecture (Tsushima, Iki, and Goto) it was close to 120 ppbv, which is the alert level for severe O_3_ pollution. Of these three remote sites, only Goto Island issued an O_x_ alert, and the present modelling system captured this high O_3_ concentration exceeding 120 ppbv well. The statistical metrics scores (see the “Methods” section for their definitions) are also shown in Fig. 4. The correlation coefficient (*R*) was from 0.61 to 0.91, the normalized mean bias (*NMB*) was from + 5.8% to + 31.2%, and the normalized mean error (*NME*) was from + 15.9% to 31.2%. Except for the grid corresponding to Nagasaki city (#3 in Fig. 4), these metrics met the model performance criteria. These evaluations of O_3_ concentration also demonstrated that the present modelling system reproduced O_3_ pollution in Nagasaki Prefecture. In addition to these validations in Nagasaki prefecture, the vertical O_3_ profiles are compared in Supplementary Fig. S4 and show that O_3_ concentration from the surface to the middle stratosphere (approximately 20 km) was also captured well by the modelling system. The NO_2_ column density observed by the satellite is compared in Supplementary Fig. S5 and a high NO_2_ concentration in the eastern part of China was generally captured. Based on these results, we confirmed the modelling system in this study captured the meteorological field and O_3_ concentration in early September 2020. The reasons for the O_x_ alert event over Goto Island despite the typhoon passing are discussed.

## Discussion

**Figure 5:**
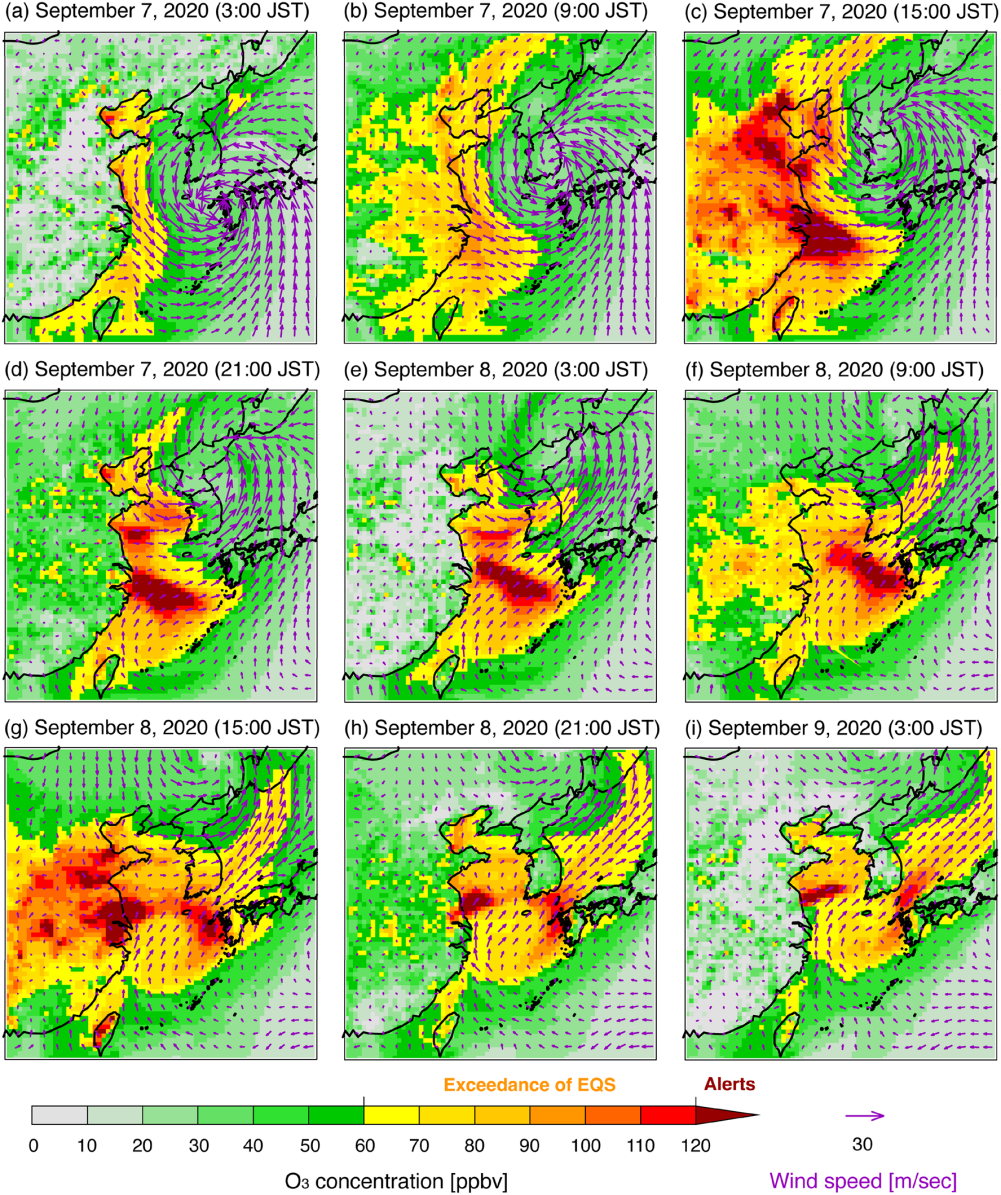
Simulated spatial distributions of O_3_ concentration at the surface level over East Asia before, during, and after the O_x_ alert on Goto Island, Nagasaki Prefecture. The maps were generated with gtool3 (http://www.gfd-dennou.org/library/gtool/index.htm.en).

To identify the reasons for the high O_3_ concentration that triggered the alert, the horizontal distribution pattern from 7 to 9 September, 2020 is shown in Fig. 5. At midnight on 7 September (Fig. 5a), high O_3_ concentration (70–90 ppbv; yellow to orange, which exceeds the Japanese EQS) was seen over the East Asian ocean adjacent to the Asian continent. In addition, the typhoon was over Goto Island, and westerly or north-westerly wind fields prevailed over the East China Sea. At this time, the O_3_ concentrations over Goto Island and other sites in Nagasaki Prefecture were low (20 ppbv; light green, see also Fig. 4). The high O_3_ concentrations stretched into the East China Sea (Fig. 5b), and O_3_ was produced during the daytime and the concentration increased on 7 September (Fig. 5c). During the night of 7 September (Fig. 5d), the high O_3_ concentration over mainland China was decreased to a low concentration by NO titration and deposition, whereas the high O_3_ concentration (120 ppbv; dark red, alert level) remained over the East China Sea. During this time, Typhoon Haishen (2020) was over the northern part of the Korean peninsula, and southerly and south-westerly wind fields transported this high O_3_ concentration to Kyushu Island (Fig. 5e and f). During the daytime on 8 September (Fig. 5g), this air mass with O_3_ higher than 120 ppbv reached Goto Island on the western edge of Kyushu Island; hence, the O_x_ alert was issued. Then, the air mass was transported to the Tsushima Strait (between Kyushu Island and the Republic of Korea) due to the south-westerly wind affected by Typhoon Haishen (2020) in Liaoning Province, northeastern China (Fig. 5h and i). This analysis of the simulated O_3_ spatial distribution suggested transboundary O_3_ transport from the Asian continent to the western part of Japan, and the wind system caused by Typhoon Haishen (2020) was important in determining the air mass transport. Based on the analysis at the top of the boundary layer (approximately 750 hPa) shown in Supplementary Fig. S5, this high transboundary O_3_ transport was limited near the surface level.

**Figure 6:**
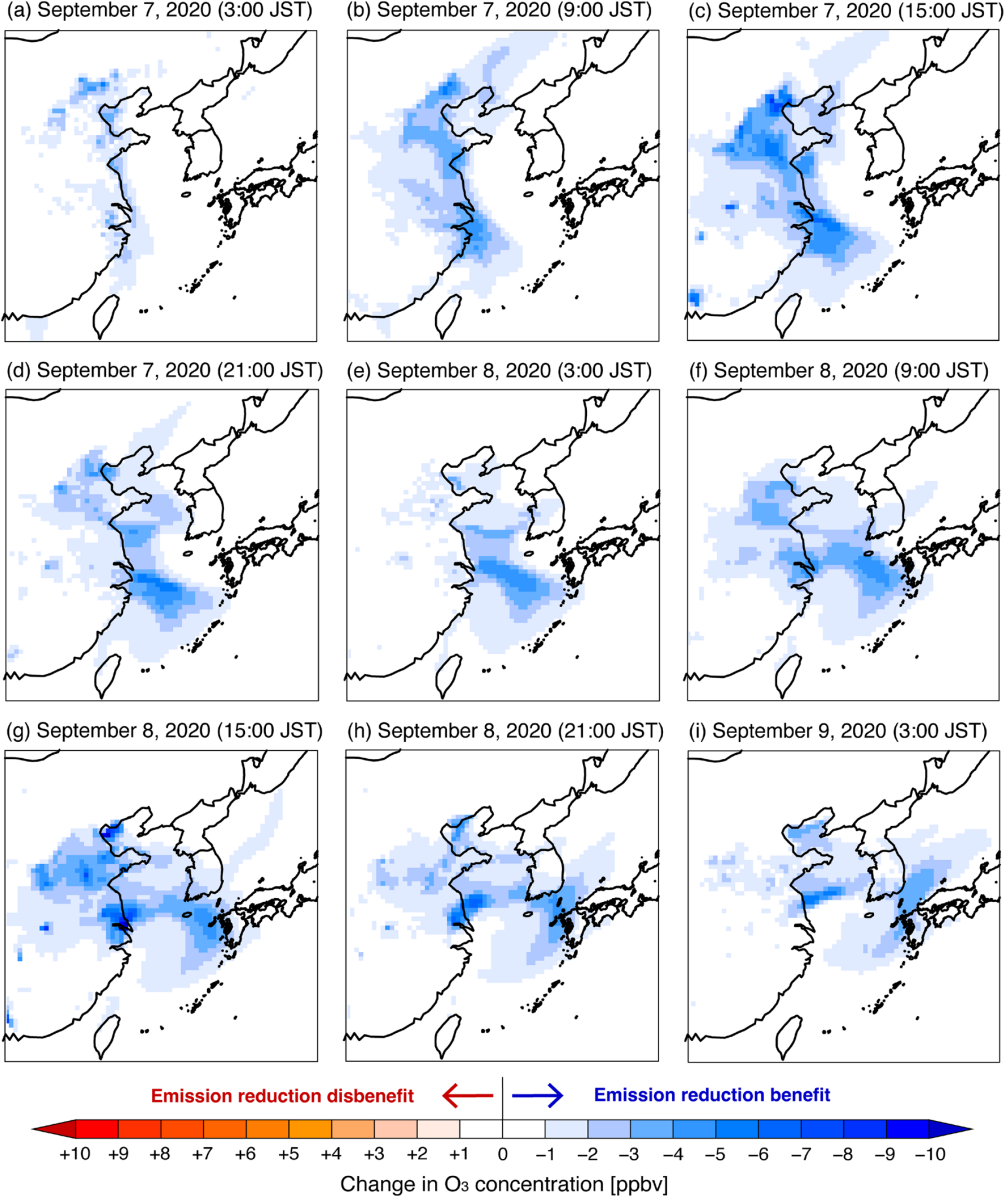
Simulated spatial distributions of change in O_3_ concentration with anthropogenic VOC emissions in China decreased by 20% ($\Delta{C}_{V}$ in Eq. (4)). Red indicates increased O_3_ concentration in the sensitivity simulation (i.e., emission reduction disbenefit), whereas blue indicates decreased O_3_ concentration (i.e., emission reduction benefit). The maps were generated with gtool3 (http://www.gfd-dennou.org/library/gtool/index.htm.en).

**Figure 7:**
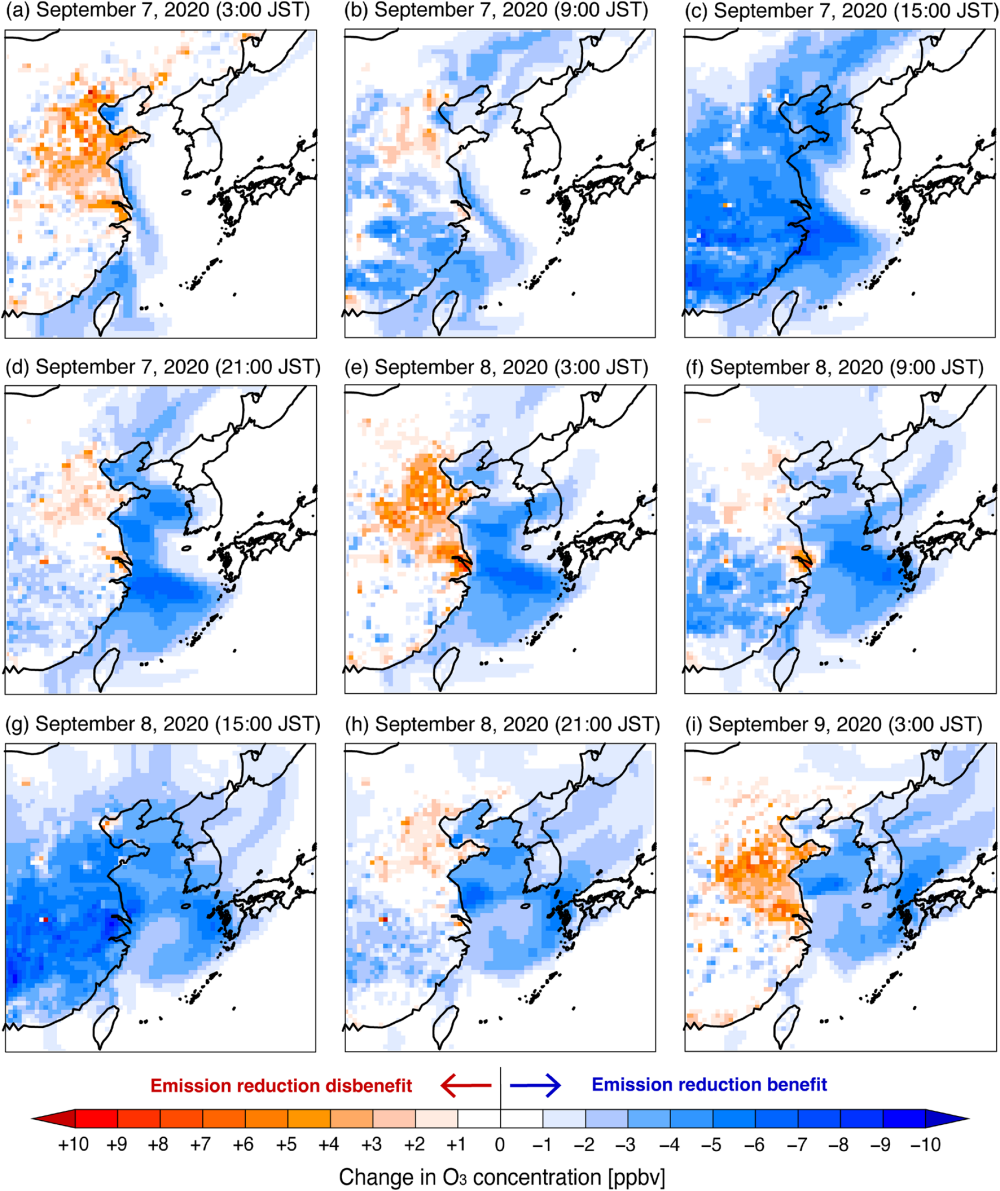
Simulated spatial distributions of change in O_3_ concentration with anthropogenic NO_x_ emissions in China decreased by 20% ($\Delta{C}_{N}$ in Eq. (5)). Red color indicates increased O_3_ concentration in the sensitivity simulation (i.e., emission reduction disbenefit), whereas blue color indicates decreased O_3_ concentration (i.e., emission reduction benefit). The maps were generated with gtool3 (http://www.gfd-dennou.org/library/gtool/index.htm.en).

The transboundary O_3_ transport after the typhoon passing was clarified, and mitigation of this severe pollution is a concern. Finally, the effects of emission regulations are discussed based on the sensitivity simulations. The changes in O_3_ concentration when 20% reductions in Chinese VOC and NO_x_ emissions ($\Delta{C}_{V}$ in Eq. (4) and $\Delta{C}_{N}$ in Eq. (5), see “Methods” section) were applied are shown in Figs. 6 and 7, respectively, and those for simultaneous VOC and NO_x_ emission reductions are shown in Supplementary Fig. S6. The reduction in anthropogenic Chinese VOC emissions always helped to reduce O_3_ concentration during the analysis period (Fig. 6). In contrast, the reduction in anthropogenic Chinese NO_x_ emissions had complex effects because it caused both O_3_ decreases and increases (Fig. 7). The increase in O_3_ was observed only over mainland China from night to early morning and was associated with the weakening NO titration effect due to the NO_x_ emission reduction. During the daytime, Chinese NO_x_ emission reduction led to an O_3_ decrease, and moreover, it always led to an O_3_ decrease over the downwind region of Japan and this impact was greater than Chinese VOC emission regulation. Therefore, NO_x_ emission regulations in China should consider these negative aspects. Recently, the aggravating effect of NO_x_ emission regulations on O_3_ pollution has been reported in China^23,24^, and the present results are also consistent with these findings.

Due to global warming, the frequency of typhoons will decrease, whereas their intensity will increase^25,26^. Similar future changes are also reported for the western North Pacific, and will lead to destructive threats by stronger wind fields and intense precipitation^27,28^. As shown in the present study, high levels of O_x_ over East Asia could be related to the meteorological fields associated with typhoons. Although precursor emission reduction could mitigate both global warming and air quality^29^, the behaviour of air pollutants related to the meteorological field should be continuously paid attention in future studies.

## Methods

### Observations

Ground-based observation of air pollutants that are regulated by EQS (carbon monoxide (CO), sulfur dioxide (SO_2_), nitrogen dioxide (NO_2_), O_x_, suspended particulate matter (SPM), and particulate matter with an aerodynamic diameter less than 2.5 μm (PM_2.5_)) are routinely measured by the Atmospheric Environmental Regional Observation System (AEROS) (https://soramame.env.go.jp). AEROS is divided into monitoring of ambient air quality by ambient air pollution monitoring stations (APMSs) and of air pollution, particularly from automobiles, by roadside air pollution monitoring stations (RAPMSs). In this study, APMSs data was used in Nagasaki Prefecture. The O_x_ measurements were obtained by the neutral-buffered potassium iodide method or ultraviolet absorption spectrometry. Although ultraviolet absorption spectrometry only detects O_3_, it was approved in 1996 as the standard method for measuring O_x_ concentrations. For the consistency of the measurement dataset, O_x_ concentrations based on the ultraviolet absorption spectrometry at APMSs were used in this study. Nagasaki Prefecture, which is the focus of this study, is on the western coast of Japan (Fig. 1). Previous studies have clarified transboundary air pollution using measurement data here because of its location, especially at remote island sites (e.g., Goto Island) where domestic emissions are small^30–32^. There are 17 sites in total in Nagasaki Prefecture, and the model grids numbered 1 to 4 (Fig. 4) include 1, 6, 5, and 2 APMSs measurements sites in the same model grid, respectively, and the averaged O_x_ concentration in the same model grid was compared with the simulation result.

In addition to the ground-based observation in Nagasaki Prefecture, vertical O_3_ profiles measured by ozonesonde distributed from the World Ozone and Ultraviolet Radiation Data Centre (WOUDC) (https://woudc.org/data/explore.php?dataset=ozonesonde) were used. During the first part of September 2020, the available ozonesonde data in East Asia were from 2 and 9 September, 2020 at King’s Park, Hong Kong (22.31° N, 114.17° E) and 3 and 8 September, 2020 at Pohang, the Republic of Korea (36.03° N, 129.38° E). A total of four vertical profile data were compared with the model result. In terms of the precursor of O_3_, satellite-derived tropospheric NO_2_ column densities can be a proxy of NO_x_ emissions^33^. The level 2 of swath measurement data taken by Ozone Monitoring Instrument (OMI) onboard the Aura was used^34^, and the information of the averaging kernel was applied for model output for fair comparison.

### Modelling simulation

To understand the horizontal distribution pattern of O_x_, the modelling simulation was conducted using the regional chemical transport model of the Community Multiscale Air Quality (CMAQ) version 5.3.3^35^. The simulation domain covering East Asia was configured by 215 × 120 grid points with a horizontal resolution of 36 km (Supplementary Fig. S2), and 44 non-uniformly spaced layers from the surface to 50 hPa with a vertical resolution of approximately 20 m for the surface layer^36^. The meteorological fields for driving CMAQ were prepared by the Weather Research and Forecasting (WRF) model version 4.4^37^. Modelling simulations were started from 8 August, 2020 and analysed from 15 August, 2020 to 14 September, 2020, discarding the first week as a spin-up period. SAPRC07tic^38^ and aero7^39^ were used for gas-phase and aerosol chemistry, respectively, and the NO_2_ aqueous-phase reaction in the SO_4_^2−^ oxidation process^40^ and better consideration of trace metal ion-catalysed O_2_ oxidation^41^ were included based on previous studies. The initial and lateral boundary conditions were prepared from autumn-averaged values of the extended CMAQ over the northern hemisphere (H-CMAQ)^42^.

The emissions inventories were prepared as follows. Anthropogenic emissions except those in China were based on Hemispheric Transport of Air Pollution (HTAP) version 3^43^. The latest available year is 2018 and this dataset with monthly variation is used. For China, the emission projections were based on the Multi-resolution Emission Inventory in China (MEIC), which includes the variation of Chinese emissions during the COVID-19 pandemic^43^. From this estimation, the anthropogenic emissions had almost recovered in August–September to the levels before the COVID-19 period, consistent with the author’s previous investigation into the transboundary PM_2.5_ transport^31^. Because the ship SO_2_ emissions were regulated according to the International Convention for the Prevention of Pollution from Ships (MARPOL) from 1 January, 2020, a reduction in SO_2_ emissions of 77% was applied based on a report by the International Maritime Organization (IMO)^45^. Biogenic emissions were prepared from the Model of Emissions of Gases and Aerosols from Nature (MEGAN)^46^ using the WRF-simulated meteorological field, and biomass-burning emissions were taken from the Global Fire Emissions Database (GFED) version 4.1^47^. SO_2_ emissions from 17 volcanoes in Japan were prepared from a measurement report by the Japan Meteorological Agency^48^.

The model performance was evaluated statistically using metrics *R*, *NMB*, and *NME*, defined as

1 $$R = \frac{{\mathop \sum \nolimits_{1}^{N} \left( {O_{i} - \overline{O}} \right)\left( {M_{i} - \overline{M}} \right)}}{{\sqrt {\mathop \sum \nolimits_{1}^{N} \left( {O_{i} - \overline{O}} \right)^{2} } \sqrt {\mathop \sum \nolimits_{1}^{N} \left( {M_{i} - \overline{M}} \right)^{2} } }} ,$$

2 $$NMB = \frac{{\mathop \sum \nolimits_{1}^{N} \left( {M_{i} - O_{i} } \right)}}{{\mathop \sum \nolimits_{1}^{N} O_{i} }} ,$$

3 $$NME = \frac{{\mathop \sum \nolimits_{1}^{N} \left| {M_{i} - O_{i} } \right|}}{{\mathop \sum \nolimits_{1}^{N} O_{i} }} ,$$

where *N* is the total number of paired observations (*O*) and models (*M*), and the corresponding averages are $\overline{O }$ and $\overline{M }$, respectively. The recommended metrics for 1-h O_3_ based on a literature review for the United States^49^ reported as the model performance goals are *R* > 0.75, *NMB* <  ± 5%, and *NME* <  + 15%, and the model performance criteria are *R* > 0.50, *NMB* <  ± 15%, and *NME* <  + 25% with no cutoff for *R* and a 40 ppbv cutoff for *NMB* and *NME*.

To estimate the change in O_3_ production from the VOC and NO_x_ precursors, sensitivity simulations that perturbed anthropogenic VOC emissions, anthropogenic NO_x_ emissions, and simultaneous anthropogenic VOC and NO_x_ emissions in China were performed in addition to the base-case simulation, which includes all emission sources. The perturbation range was a 20% emission reduction from the base-case, which is typical for potential emission controls^8^. The change in O_3_ concentration from the base-case simulation was calculated based on the following three definitions. In this study, the changes in concentration were directly shown and not scaled to be expressed as sensitivities.

4 $${\Delta }C_{V} { = } C_{{{\text{Chinese VOC emission }}20{\text{\% reduction case}}}} - C_{{{\text{Base}} - {\text{Case}}}} ,$$

5 $${\Delta }C_{N} { = }C_{{{\text{Chinese NOx emission }}20{\text{\% reduction case}}}} - C_{{{\text{Base}} - {\text{Case}}}} ,$$

6 $${\Delta }C_{NV} { = } C_{{{\text{Chinese VOC and NOx emission }}20{\text{\% reduction case}}}} - C_{{{\text{Base}} - {\text{Case}}}} ,$$

### Supplementary Information

Supplementary Figures.

## Data Availability

The datasets generated during and/or analysed during the current study are available from the corresponding author on reasonable request.
