# Supplementary material for: Severe level of photochemical oxidants (Ox) over the western coast of Japan during autumn after typhoon passing
Source: Sci Rep. 2023 Sep 29;13:16369. doi: 10.1038/s41598-023-43485-0 (PMC10541868; doi:10.1038/s41598-023-43485-0)
Supplement: Supplementary file 1 — Supplementary Figures. [file 41598_2023_43485_MOESM1_ESM.pdf]

Supplementary Information

Severe level of photochemical oxidants (O_x) over the western coast of Japan during autumn after typhoon passing

Syuichi Itahashi

Sustainable System Research Laboratory (SSRL), Central Research Institute of Electric Power Industry (CRIEPI), Abiko, Chiba 270-1194, Japan; isyuichi@criepi.denken.or.jp

Contents

Supplementary Figures S1–S7

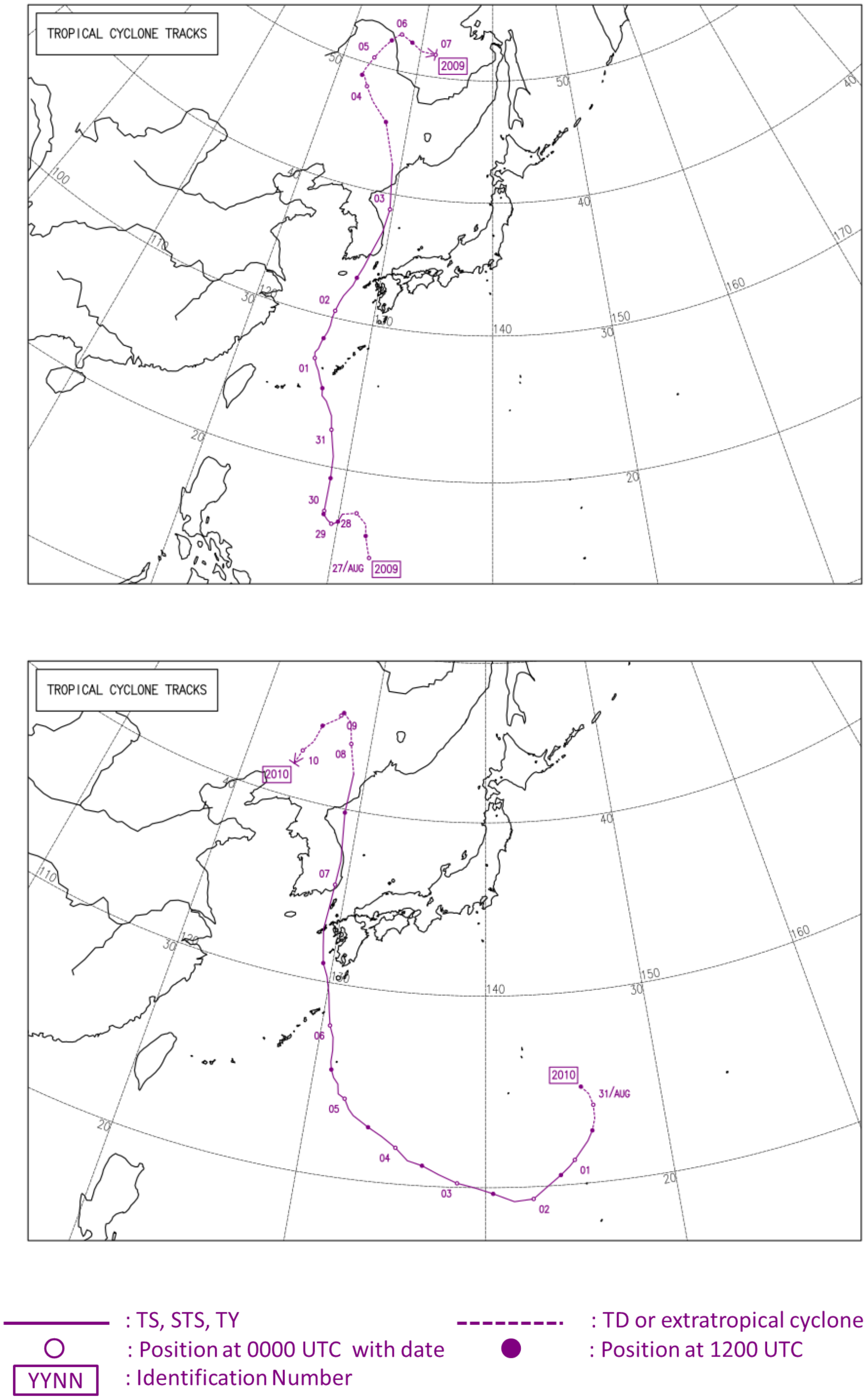

Supplementary Figure S1. Japan Meteorological Agency (JMA) best tracks for (top) Typhoon Maysak (2020) and (bottom) Typhoon Haishen (2020). The figures are available from https://www.jma.go.jp/jma/jma-eng/jma-center/rsmc-hp-pub-eg/bstve_2020_m.html.

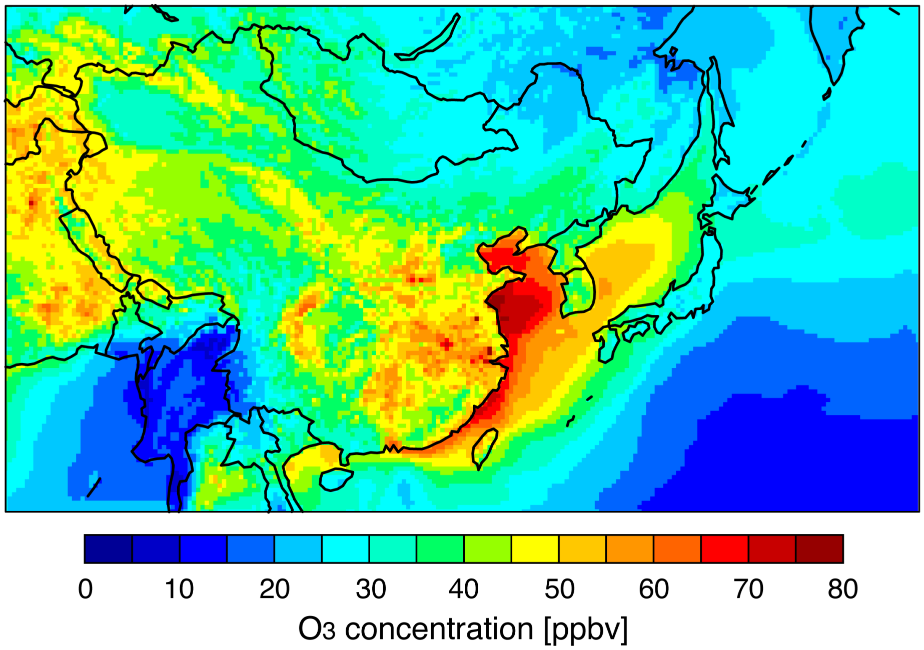

Supplementary Figure S2. Modelling domain with a 2-week average (from September 1 to September 14, 2020) simulated O_3 concentration. The maps were generated with gtool3 (http://www.gfd-dennou.org/library/gtool/index.htm.en).

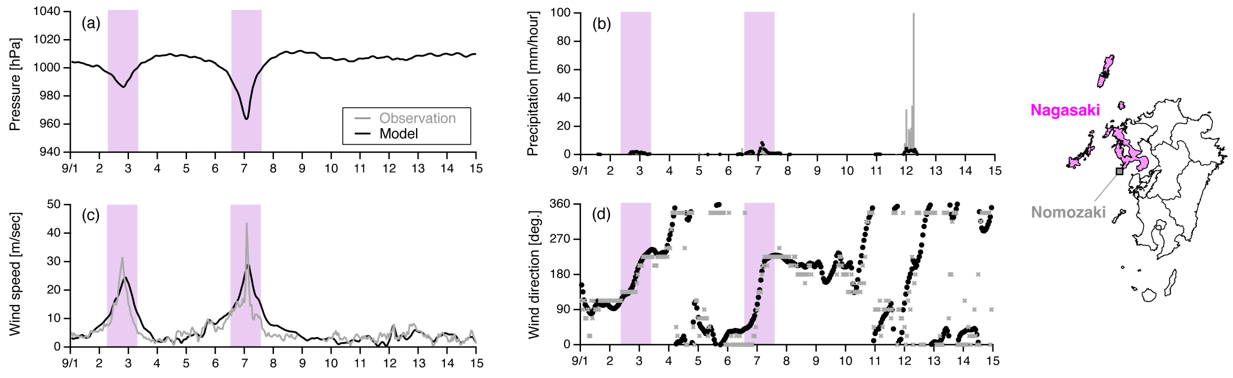

Supplementary Figure S3. Comparison of the meteorological fields of (a) pressure, (b) precipitation, (c) wind speed, and (d) wind direction for observations (grey) and the model (black) at Nomozaki, AMeDAS station in Nagasaki. Note that there is no observation for (a) pressure at this site. The purple shading indicates the days affected by Typhoon Maysak (2020) and Typhoon Haishen (2020). The maps were generated with GMT (https://docs.generic-mapping-tools.org/dev/index.html).

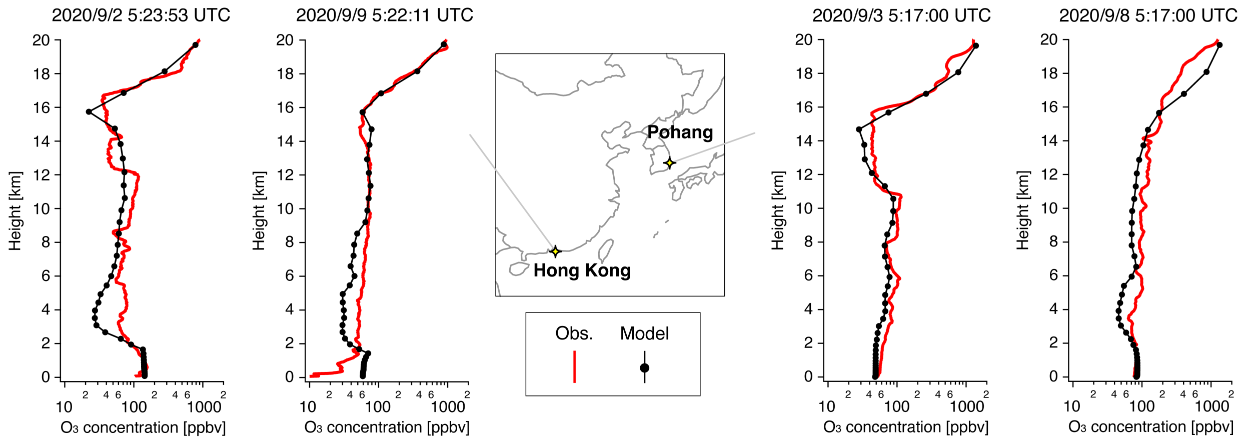

Supplementary Figure S4. Vertical profiles of O_3 concentration for observations (red) and the model (black) at Asian ozonesonde sites in (left) Hong Kong and (right) Pohang, the Republic of Korea. The maps were generated with GMT (https://docs.generic-mapping-tools.org/dev/index.html).

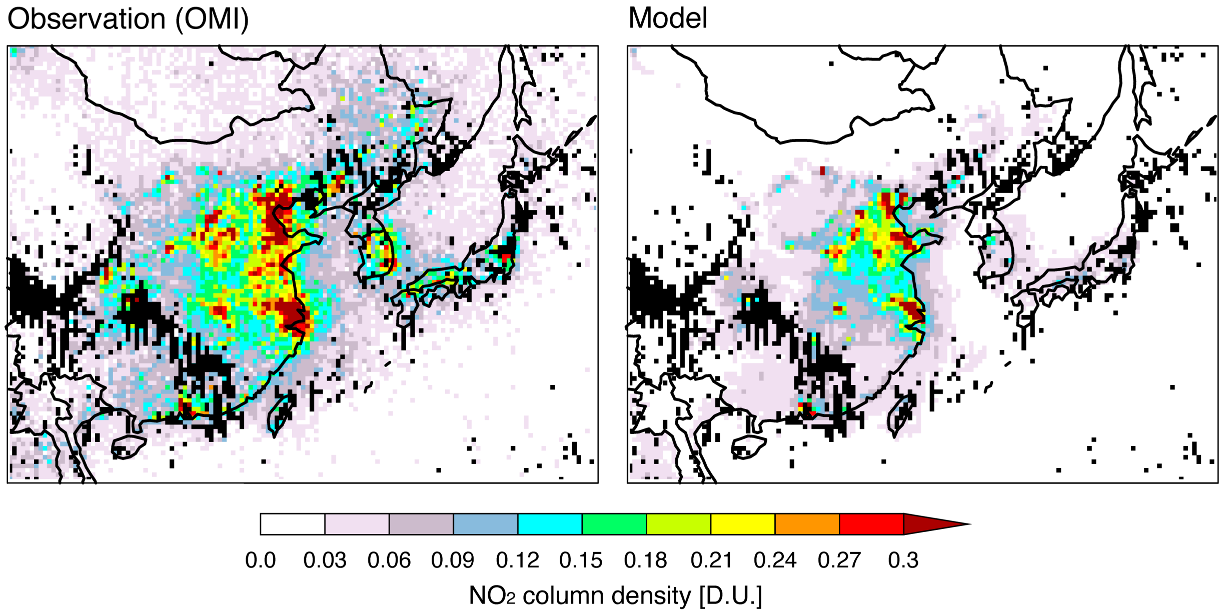

Supplementary Figure S5. Comparison of NO_2 column density between (left) observation of OMI satellite and (right) the model averaged over 2-week (from September 1 to September 14, 2020). The black dotted indicates the data deficit. The maps were generated with gtool3 (http://www.gfd-dennou.org/library/gtool/index.htm.en).

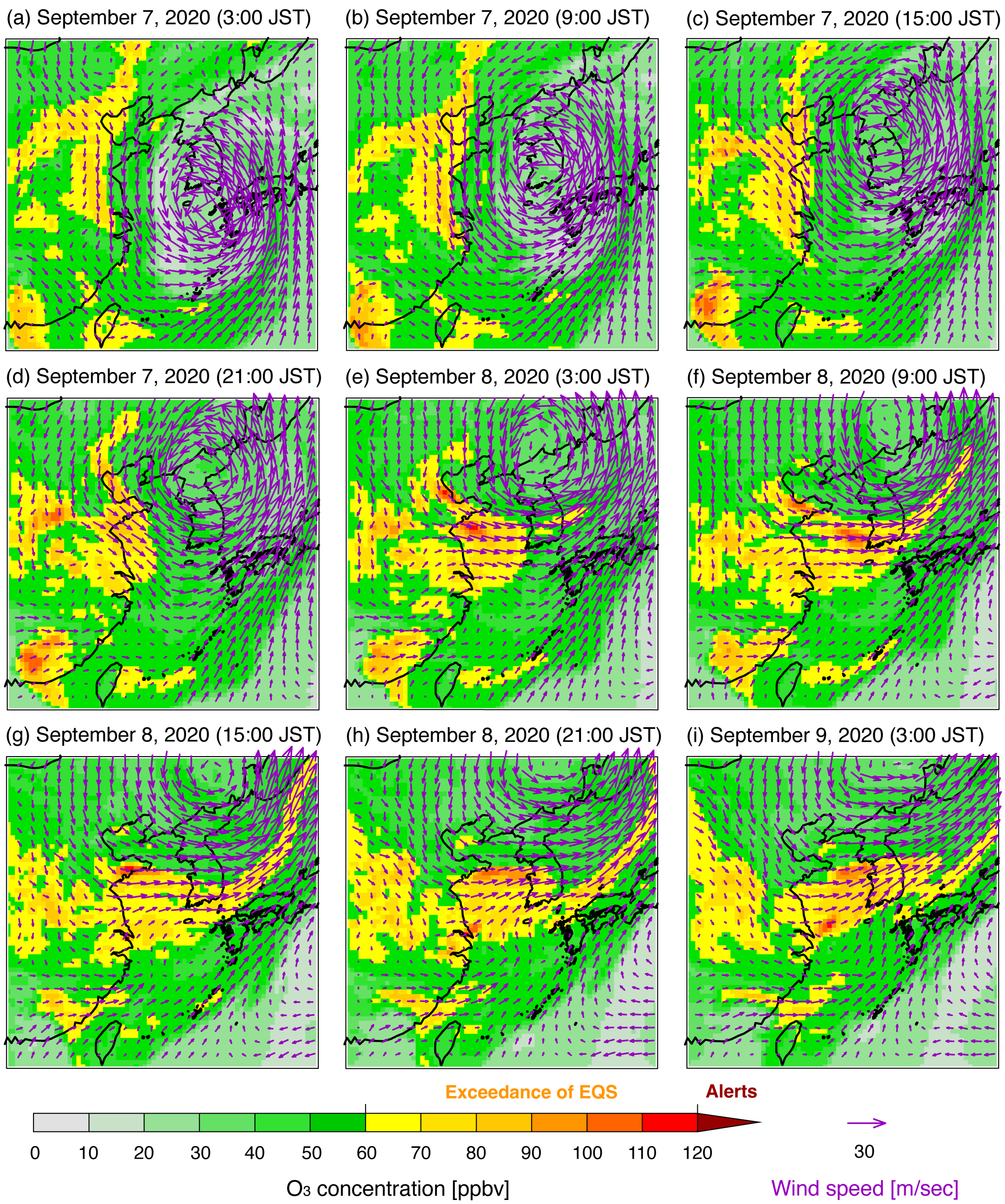

Supplementary Figure S6. Simulated spatial distributions of O_3 concentration at the top of the boundary layer (approximately 750 hPa) over East Asia before, during, and after the O_x alert on Goto Island, Nagasaki Prefecture. The maps were generated with gtool3 (http://www.gfd-dennou.org/library/gtool/index.htm.en).

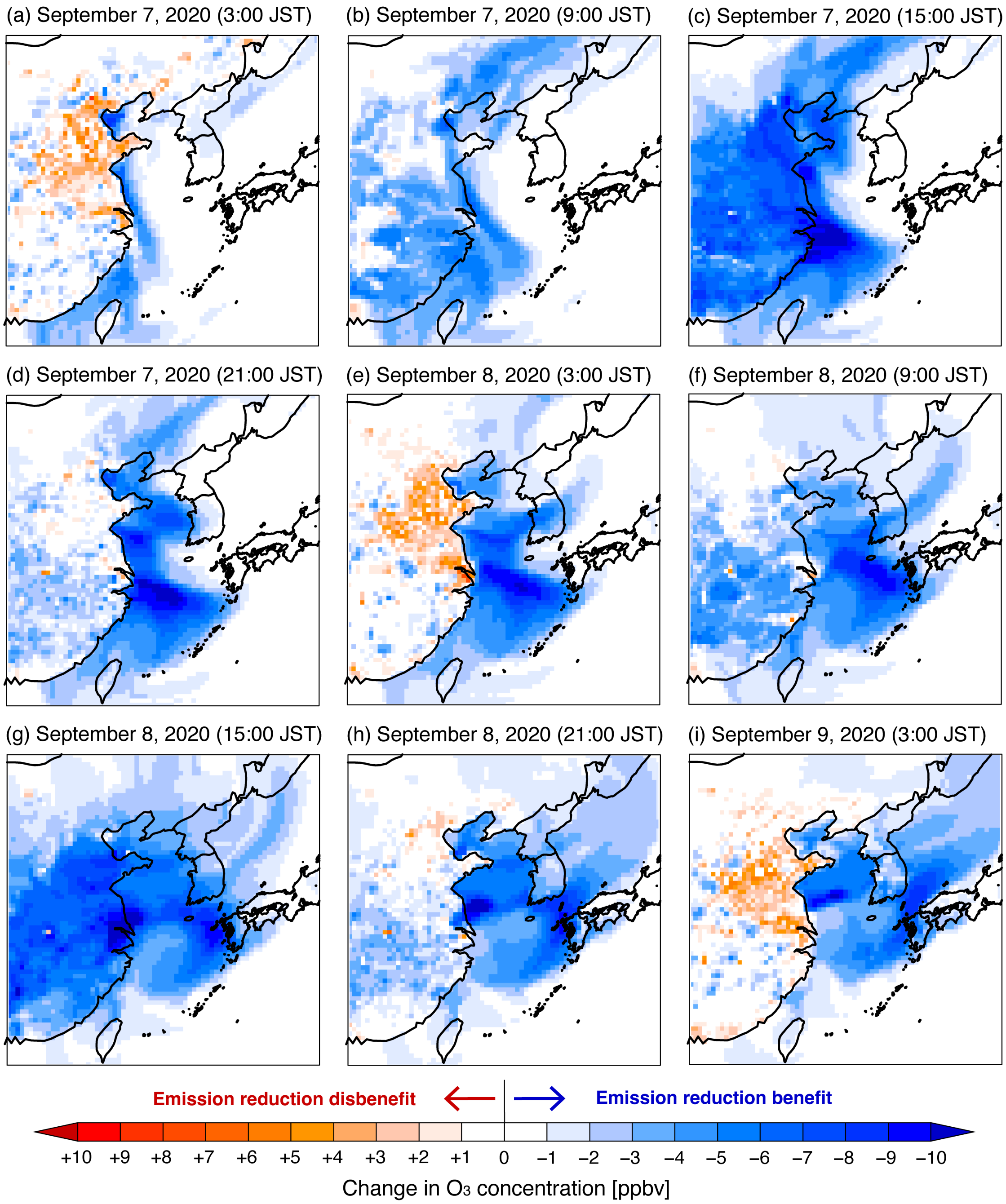

Supplementary Figure S7. Simulated spatial distributions of change in O_3 concentration with anthropogenic NO_x and VOC emissions in China decreased by 20% (ΔC_{NV} in equation (6)). Red color indicates increased O_3 concentration in the sensitivity simulation (i.e., emission reduction disbenefit), whereas blue color indicates decreased O_3 concentration (i.e., emission reduction benefit). The maps were generated with gtool3 (http://www.gfd-dennou.org/library/gtool/index.htm.en).
